# Decoding post-transcriptional gene expression controls in trypanosomatids using machine learning

**DOI:** 10.12688/wellcomeopenres.23817.1

**Published:** 2025-04-04

**Authors:** Michele Tinti, David Horn

**Affiliations:** 1 The Wellcome Centre for Anti-Infectives Research, Biological Chemistry & Drug Discovery, University of Dundee Division, Dundee, Scotland, UK

**Keywords:** UTRs, Codon Bias, Trypanosoma, Leishmania, Machine Learning, Translation Efficiency

## Abstract

**Background:**

We recently described a pervasive cis-regulatory role for sequences in *Trypanosoma brucei* mRNA untranslated regions (UTRs). Specifically, increased translation efficiency (TE) was associated with the dosage and density of A-rich tracts. This finding raised three related questions: (1) What relative contributions do UTRs and codon usage bias make to TE in *T. brucei*? (2) What relative contributions do these sequences make to mRNA steady-state levels in *T. brucei*? (3) Do these sequences make substantial contributions to TE and/or mRNA steady-state levels in the related parasitic trypanosomatids, *T. cruzi* and *Leishmania*?

**Methods:**

To address these questions, we applied machine learning to analyze existing transcriptome, TE, and proteomics data.

**Results:**

Our predictions indicate that both UTRs and codon usage bias impact gene expression in all three trypanosomatids, but with substantial differences. In *T. brucei*, TE is primarily correlated with longer A-rich and C-poor UTRs. The situation is similar in *T. cruzi*, but codon usage bias makes a greater contribution to TE. In *Leishmania*, median TE is higher and is more strongly correlated with longer (A)U-rich UTRs and with codon usage bias. Codon usage bias has a major impact on mRNA abundance in all three trypanosomatids, while analysis of *T. brucei* proteomics data yielded results consistent with the view that this is due to differential translation elongation rates.

**Conclusions:**

Taken together, our findings indicate that gene expression control in trypanosomatids operates primarily at the point of translation, which is impacted by both UTRs and codon usage. We suggest a model whereby UTRs control the rate of translation initiation, while favoured codons increase the rate of translation elongation, thereby reducing mRNA turnover.

## Plain Language Summary

We study how three parasites ( *Trypanosoma brucei*, *Trypanosoma cruzi*, and *Leishmania*) control gene expression. Using computer analyses, we looked at two key factors: alternative codons, which are translated to incorporate the same amino acid in a protein, and UnTranslated Regions (UTRs); both of which can impact messenger RNA stability or the rate at which messenger RNA is translated to produce protein. We found that the impact of codons and UTRs is primarily at the point of translation. Codon usage bias likely impacts mRNA stability by increasing the rate of translation. Understanding these regulatory processes will reveal how these parasites and related cells function, in terms of expressing thousands of different proteins at appropriate levels.

## Introduction

Trypanosomatids are a group of protozoan parasites that cause severe diseases in humans and animals, including African sleeping sickness ( *Trypanosoma brucei*), Chagas disease ( *Trypanosoma cruzi*), and visceral leishmaniasis ( *Leishmania donovani* and *L. infantum*) ^1^. These diseases are associated with significant morbidity and mortality, particularly in low-income regions of the world, making trypanosomatids important targets for biomedical research. These parasites also present models for studying gene expression mechanisms ^2^, and these studies have the potential to facilitate the development of new therapeutic strategies ^3, 4^.

Gene expression in trypanosomatids is unusual compared to most eukaryotes, as it relies on polycistronic transcription, where multiple unrelated genes are transcribed as a single unit and processed to yield individual mRNAs ^2^. This unconventional mechanism limits the ability of these parasites to regulate gene expression at the level of transcription, a key regulatory step in most organisms. Instead, trypanosomatids rely heavily on post-transcriptional mechanisms, including mRNA maturation, turnover and translation efficiency (TE), to control gene expression ^2, 5^. TE has been experimentally measured in trypanosomatids by calculating the ratio between ribosome footprint densities, assessed using Ribo-seq, and mRNA levels, assessed by RNA-seq ^6– 8^. Post-transcriptional expression can be influenced by several factors, including codon usage bias, the preference for certain codons over others encoding the same amino acid, and regulatory elements within mRNA untranslated regions (UTRs), located at both the 5' and 3' ends of mRNA. Codon usage bias impacts both mRNA abundance and TE in *T. brucei* ^9, 10^, while thousands of UTRs were recently reported to primarily impact TE in *T. bruce*i ^11^.

Although both codon usage and UTRs impact gene expression in eukaryotes ^12– 14^, their relative contributions to mRNA abundance and TE remain unclear. Given the likely complex relationships between sequence features, mRNA abundance and TE, as well as the number of potential regulatory sequence features involved, we hypothesized that a machine learning approach would be particularly well-suited to decoding these regulatory patterns and their interactions. To test this hypothesis, we developed a machine learning framework to investigate the contributions of codon bias and UTRs to previously published measures of mRNA abundance and TE in *T. brucei* ^7^, *T. cruzi* ^6^ and *L. donovani* ^8^ as well as measures of protein abundance in *T. brucei*. Our approach allowed us to quantify the relative importance of different regulatory features in determining mRNA abundance and TE in all three parasites.

## Methods

### Translation efficiency data

To quantify TE across trypanosomatid species, we reanalyzed previously published RNA-seq and ribosome profiling datasets for *T. brucei*, *T. cruzi*, and *L. donovani* ^6– 8^. For *T. brucei* (TREU927) bloodstream and procyclic forms and *L. donovani* (BPK282A1) promastigote forms, raw sequencing data were processed through a standardized pipeline: initial quality control was performed using FastQC ( https://www.bioinformatics.babraham.ac.uk/projects/fastqc/), followed by adapter trimming and quality filtering with Fastp (0.20.0) ^15^. Processed reads were aligned to the respective reference genomes (TriTrypDB v68) ^16^ using Bowtie2 (2.3.5) ^17^ with '--very-sensitive-local' parameters. The resulting alignments were processed with SAMtools (1.9) ^18^ for sorting and indexing, and PCR duplicates were marked using Picard MarkDuplicates (2.22.3) ^19^. Read counts per coding sequence were quantified using featureCounts (1.6.4) ^20^ with parameters accounting for multi-mapping reads (-M) and overlapping features (-O). For *T. cruzi* (Dm28c2018) epimastigote forms, due to the colour-space nature of the SOLiD sequencing data, we employed a modified pipeline where reads were aligned using SHRiMP2 (2.2.3) ^21^, specifically designed for colour-space reads, followed by featureCounts quantification. For all species, raw counts were normalized to TPM using the formula TPM = (10^6 * (C/L)) / sum(Cg/Lg) where C represents the number of reads mapped to a gene, L is the gene length in base pairs, Cg and Lg refer to the counts and length of each gene g. For downstream analyses, we calculated two key metrics: TE and mRNA abundance. TE was computed as the ratio between ribosome footprint TPM and total TPM (defined as ribosome footprint TPM + mRNA TPM) for each gene, yielding values between 0 and 1. mRNA abundance was quantified using the log10-transformed mRNA TPM values.

### UTR sequences

To derive 5' and 3' UTR sequences for *T. brucei*, *T. cruzi*, and *L. donovani*, we analyzed polycistronic transcript structures using genome annotations from TriTrypDB version 68 ^16^. For each species, we downloaded GFF files containing genome feature coordinates and systematically analyzed inter-CDS regions. The 3' UTR sequences were computationally extracted from the intergenic regions between consecutive coding sequences (CDS) in the same transcriptional orientation using a Python script. Specifically, for each gene, its 3' UTR was defined as the intergenic region between its stop codon and 200 nucleotides upstream of the start codon of the downstream gene. The 5' UTRs were defined as the first 150 nucleotides upstream of each CDS start codon. To ensure data quality and consistency, we only retained genes in our analysis for which both 5' and 3' UTRs could be determined based on these criteria.

### Feature extraction

Feature extraction from 5' and 3'-UTRs was performed as previously described ^11^ using a Python script that harnessed the ‘re’ module for regular expression pattern matching and Biopython ^22^ for sequence manipulation. Our script analyzed various DNA sequence attributes, such as nucleotide base counts and the prevalence of poly-purine, poly-pyrimidine, and homopolymeric stretches. These features were normalized against the total length of each 3'-UTR. DNA sequence features were extracted using a standardized naming convention: region identifiers for 3’ and 5’ UTRs (3utr_ or 5utr_) precede feature types, which include base frequencies (c_) and tract patterns (ct_) of individual nucleotides (A/T/G/C) or their combinations (AT/CG/CT/AG). Tract patterns are annotated with mismatch tolerances (_m0, _m1, or m2, indicating 0-2 allowed mismatches). For coding sequences (CDS), nucleotide composition was analyzed by calculating the normalized frequency of each base (A, T, G, C) at the third codon position, denoted by the prefix third_base. UTRs features were normalized by their respective sequence lengths to account for size variation. CDS frequencies were normalized by the total number of codons for gene size variation. To assess codon usage optimization, we leveraged the codon frequency tables available at TriTrypDB to identify non-optimal codons. A codon was considered non-optimal when its frequency was lower than the most abundant codon encoding the same amino acid. For each CDS, we then calculated a non-optimal codon usage score (non_opt_codon) by counting the occurrences of non-optimal codons and normalizing by the total number of codons in the sequence.

### Machine learning

We analyzed genes with expression levels above a minimum threshold of 5 TPM in both RNA-seq and ribosome profiling datasets. To minimize redundancy and potential biases from highly similar sequences, we performed clustering analysis on the first 200 nucleotides of 3' UTR sequences. Sequence clustering was performed using MMseqs2 (13.45111) ^23^ with parameters --min-seq-id 0.4, removing genes with highly similar UTR regions (sequence identity > 40%). Our dataset was partitioned into a 70% training set and a 30% test set in a 3-fold cross-validation. To address multicollinearity, we implemented a recursive correlation-based feature elimination method. Starting with a complete correlation matrix, we iteratively removed features showing correlations above 0.95 with other features, recalculating the correlation matrix after each removal until no remaining features showed correlations above this threshold. We assessed the models using Spearman's rank correlation coefficient (SRCC) calculated on the out-of-fold predictions. We also evaluated the models using 100 independent train-test splits with different random seeds and visualized the distribution of the resulting SRCC values. Finally, to interpret the Random Forest model, we visualized the importance of the top twenty features using (SHapley Additive exPlanations) SHAP values ^24^ and the third out of fold set, thereby elucidating the contribution of each UTR and CDS feature to the TE and mRNA abundance predictions. To visualize feature interactions, we used the dependence plot function, which automatically identified the strongest interacting feature with UTR length based on the highest SHAP interaction score. SHAP values and SHAP interaction scores were computed with the shap Python package v0.35.

### Protein abundance

For proteomic analysis, we obtained intensity-based absolute quantification (iBAQ) data for *T. brucei* bloodstream form ^25^. To ensure data quality and avoid redundancy in cases of multiple protein isoforms, we selected the first listed protein from each protein group for analysis. The dataset was filtered to include only proteins with iBAQ values greater than 10^4, establishing a threshold that ensures reliable quantification. These filtered protein abundance data were then subjected to the same machine learning framework described above.

## Results

### Differences in expression and base-composition profiles

**Figure 1.**
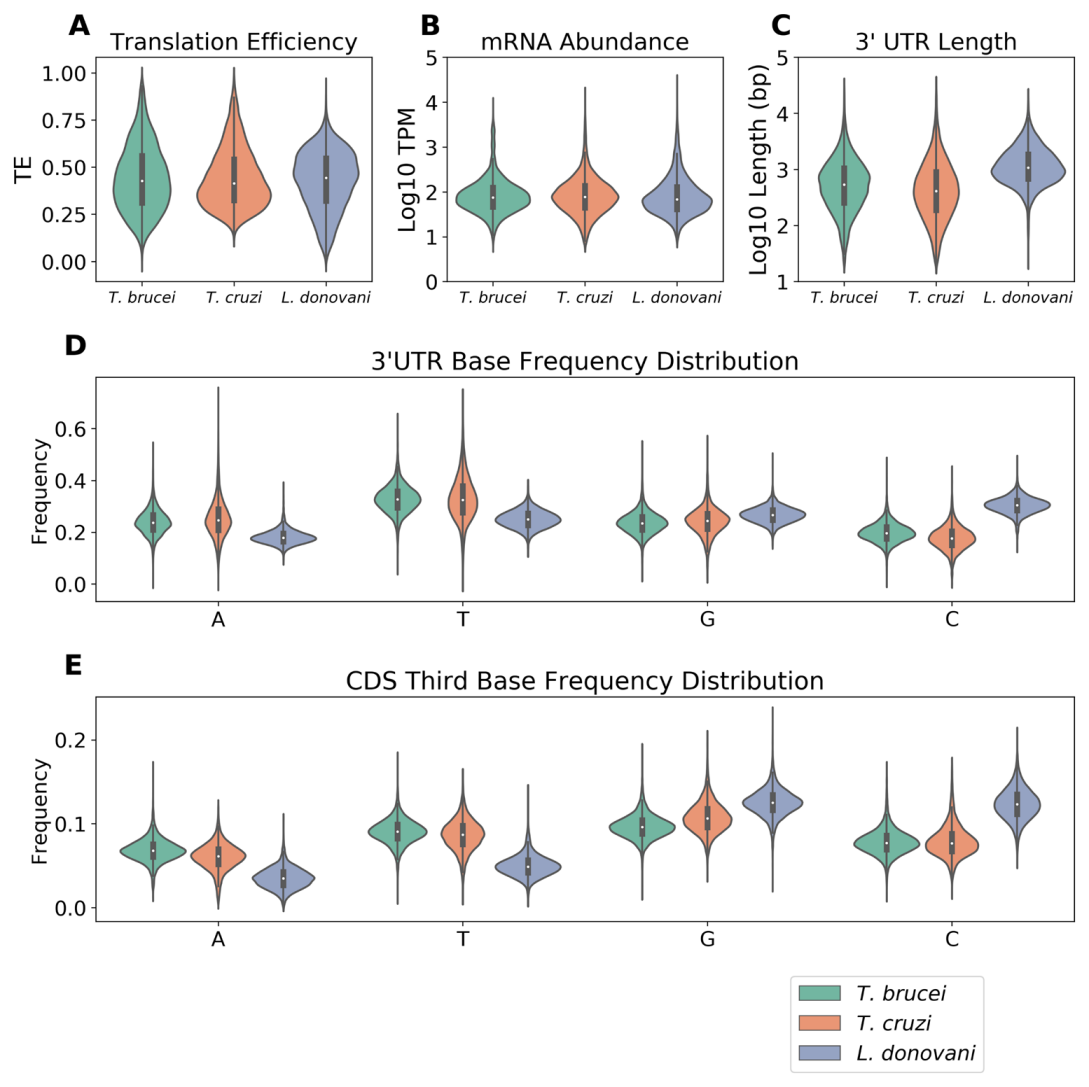
Differences in expression and base-composition profiles. **A** The violin plot shows TE value distributions. TE was calculated as the ratio between ribosome footprint TPM and total TPM (ribosome footprint TPM + mRNA TPM), resulting in values between 0 and 1. *T. brucei* (green, n = 6923), *T. cruzi* (orange, n = 6800), and *L. donovani* (blue, n = 6026). **B** The violin plot shows the distribution of mRNA abundance values. **C** The violin plot shows the distribution of mRNA lengths. **D** The violin plot shows the frequency distribution of each nucleotide (A, T, G, C) in 3'-UTRs of *T. brucei*, *T. cruzi*, and *L. donovani*. **E** The violin plot shows the nucleotide frequencies at the third codon position in coding sequences of *T. brucei*, *T. cruzi*, and *L. donovani.* For all violin plots, the internal black bars indicate median and interquartile range.

To investigate post-transcriptional expression controls in trypanosomatids, we first analyzed experimental measurements of gene expression in *T. brucei* ^7^, *T. cruzi* ^6^ and *L. donovani* ^8^. Translation efficiency (TE) distributions revealed distinct profiles among the three species ( Figure 1A). *L. donovani* exhibited the highest median TE value, *T. cruzi* displayed the lowest, while *T. brucei* showed the widest distribution. In contrast, mRNA abundance distributions were similar across species ( Figure 1B), with comparable median expression levels (log10 TPM values between 1.5 and 2.0). We then examined sequence properties that might influence these expression patterns. Analysis of 3'-UTR sequences, inferred from intergenic regions (see Methods), revealed that *L. donovani* possesses distinctly longer UTRs, with a median length of 1075 b compared to 558 b in *T. brucei* and 411 b in *T. cruzi* ( Figure 1C). Base composition analysis highlighted another unique feature of *L. donovani*: its 3'-UTRs display higher G/C and lower A/T content compared to the trypanosomes ( Figure 1D). This G/C bias extends to coding sequences, where *Leishmania*, as reported previously ^26^, shows elevated G/C content at the third codon position compared to both *T. brucei* and *T. cruzi* ( Figure 1E).

### Species-specific contributions of UTRs and codon bias to translational control

**Figure 2.**
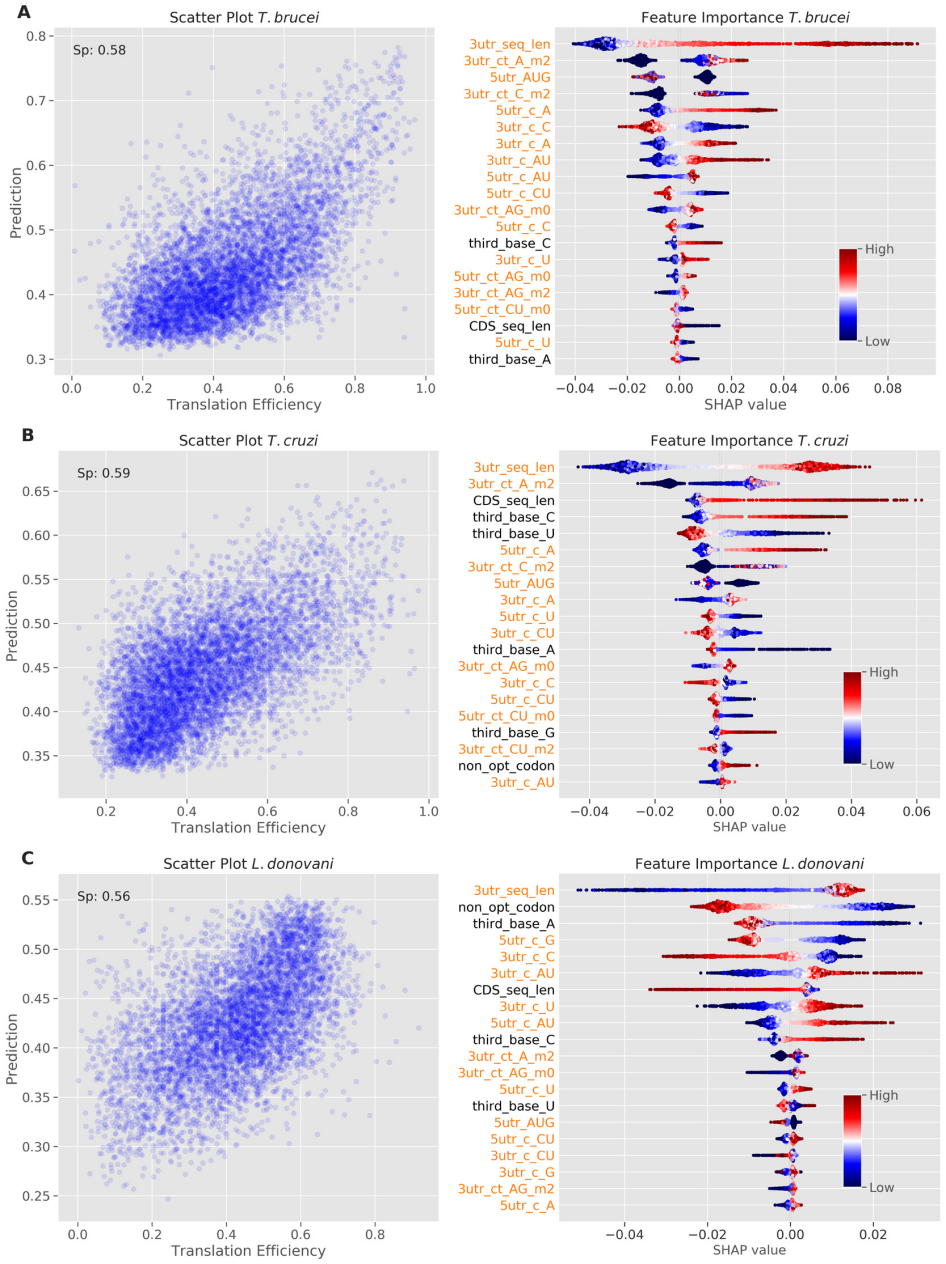
Machine learning models reveal determinants of translation efficiency. Model performance and feature importance analysis for **A** *T. brucei*. **B** *T. cruzi*. **C** *L. donovani*. The scatter plots on the left show prediction versus measured TE values. The Spearman's rank correlation coefficient (Sp) is reported for each model. The beeswarm plots on the right show SHapley Additive exPlanations (SHAP) values; red indicates high feature value and blue indicates low feature value, with the magnitude of SHAP values indicating the strength and direction of each feature's impact on model predictions. Features are ordered by their absolute SHAP values (the sum of each point's absolute value), with the most important features at the top. Dots are jittered in the y-axis to illustrate SHAP values distribution. The lengths of CDS sequences and predicted 3' UnTranslated Regions (UTRs) are captured by ‘CDS_seq_len’ and '3utr_seq_len' features respectively. Features extracted from UTR regions are colour-coded in orange, with '3utr_' indicating 3' UTR features and '5utr_' indicating 5' UTR features. Simple base frequencies are denoted by the prefix 'c_' while tract frequencies use 'ct_'. Tract features are suffixed with '_m0', '_m1', or '_m2' to indicate the number of allowed mismatches (0, 1, or 2 respectively) between consecutive nucleotide stretches. Individual nucleotide frequencies at the third position of codons in coding sequences are denoted by the prefix 'third_base'. The frequency of non-optimal codons in coding sequences is represented by the 'non_opt_codon' feature.

To identify and rank sequence features that influence TE, we developed machine learning models for *T. brucei*, *T. cruzi*, and *L. donovani*. Briefly, we employed the random forest regression strategy described in 11, but now extracting sequence features from both the CDS and UTR sequences. All models showed predictive power for TE with Spearman's rank correlation coefficient values above 0.55 ( Figure 2). Feature importance and their contributions to the predictions were visualized using the SHAP (SHapley Additive exPlanation) game theory-based method; the SHAP values for the top twenty features are shown in Figure 2.

The analysis revealed contributions from both UTRs and codon usage bias to TE control in all three trypanosomatids, but with different relative impacts. In *T.* brucei ( Figure 2A), 3'-UTR sequence length emerged as the strongest predictor of increased TE (indicated in red), followed by 3'-UTR A-rich tract count (3utr_ct_A_m2). This is consistent with our recent finding that the dosage and density of A-rich poly-purine tracts within 3’-UTRs correlated with TE ^11^. While the model indicated that similar 5'-UTR sequence features also contribute to increased TE, codon bias made a lesser contribution. Nevertheless, C at the third position was positively correlated with TE, while A at the third position was negatively correlated with TE ( Figure 2A), as reported previously ^9, 10^.

The *T. cruzi* model showed a similar distribution of features that contribute to TE control, also registering 3'-UTR sequence length and 3'-UTR A-rich tracts as the strongest predictors of increased TE ( Figure 2B). In this case though, the importance of codon bias in TE control was increased relative to *T. brucei*; positive correlation with C at the third position and negative correlation with U at the third position ranked fourth and fifth in importance, respectively ( Figure 2B). This suggests that codon usage bias has a greater impact on TE control in *T. cruzi* than in *T. brucei*, albeit with UTRs having the greatest impact in both species.

For the *L. donovani* model, 3'-UTR sequence length, once again, emerged as the strongest predictor of TE, followed by non-optimal codon usage as a predictor of reduced TE ( Figure 2C). The UTR sequences contributing to TE in *Leishmania* were similarly C-poor, but distinct from the trypanosomes in that they were (A)U-rich. Optimal codons contributing to TE were similar among the trypanosomatids. An upstream open reading frame (5utr_AUG) had a negative impact on TE in all three trypanosomatids, as expected ^27, 28^, while longer CDSs registered a positive impact on TE in *T. cruzi* but not in the other trypanosomatids.

**Figure 3.**
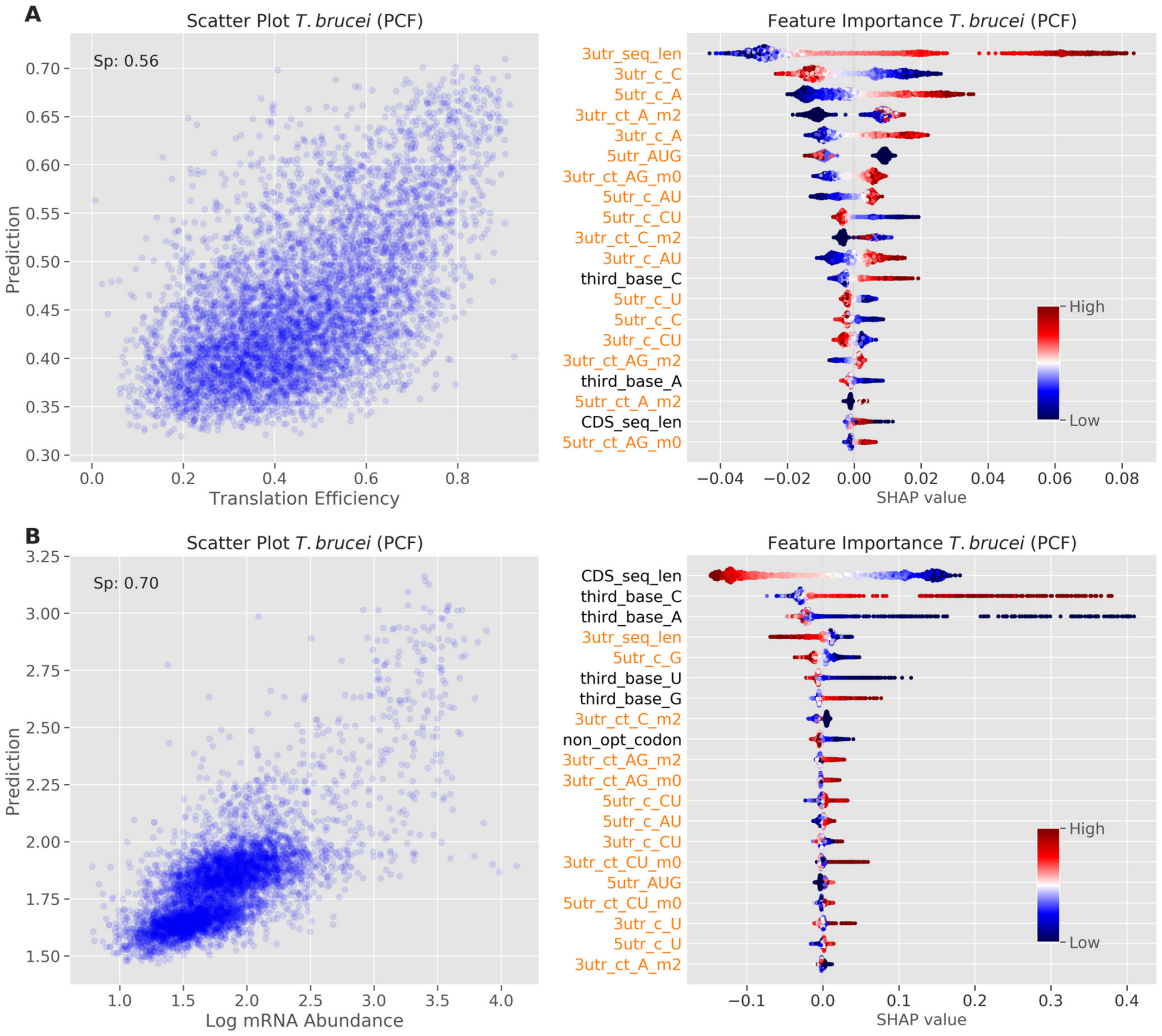
Machine learning prediction and feature importance analysis for the insect stage of *T. brucei*. Model performance (left) and feature importance analysis (right) for insect stage *T. brucei*. **A** TE. The scatter plot on the left shows prediction versus measured TE values. **B** mRNA abundance. The scatter plot on the left shows prediction versus measured mRNA abundance values (log10 transformed). Other details as in Figure 2.

We suspected that the patterns observed above represented species differences rather than developmental differences within a species, since a relatively small proportion of transcripts and proteins display substantial developmental regulation in trypanosomatids. As a test of this hypothesis, we ran the machine learning analysis on expression data from insect stage *T. brucei* ^7^, and compared the results ( Figure 3A) to the bloodstream stage analysis ( Figure 2A). Feature importance and their relative contributions to the TE predictions, as visualized using SHAP values, were very similar for these two distinct life cycle stages, as expected. We conclude that the patterns observed above do indeed represent species differences rather than developmental differences within a species.

**Figure 4.**
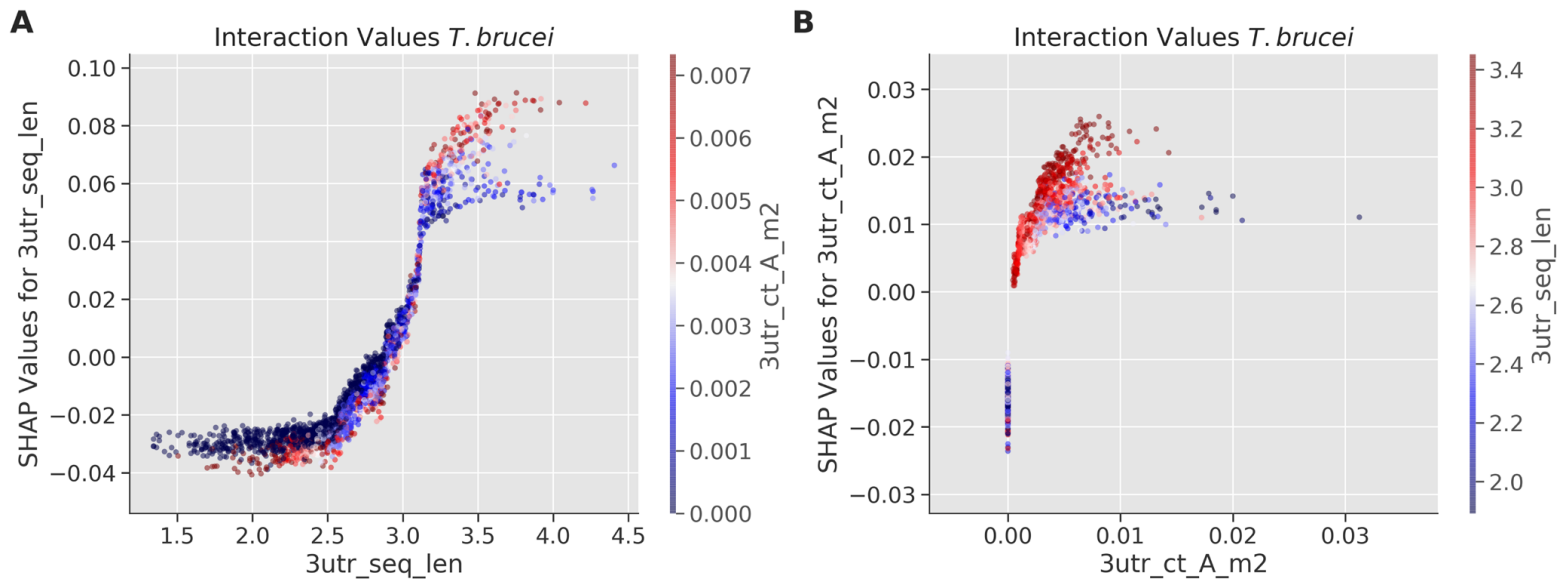
Interaction between 3'-UTR sequence length and A-rich tracts in *T. brucei*. Feature interaction analysis. The SHapley Additive exPlanations (SHAP) interaction plots illustrate the relationship between 3'-UTR sequence length (3utr_seq_len) and A tract frequency (3utr_ct_A_m2). **A** The y-axis represents the SHAP values for 3'-UTR sequence length, indicating its contribution to model predictions. The x-axis represents 3'-UTR sequence length in log scale. Points are coloured according to the value of the top interacting feature (3utr_ct_A_m2), with red indicating high values and blue indicating low values. **B** The y-axis represents the SHAP values for A tract frequency (3utr_ct_A_m2), indicating the contribution to model predictions. The x-axis represents A tract frequency values. Points are coloured according to the value of the top interacting feature (3utr_seq_len), with red indicating high values and blue indicating low values. Other details as in Figure 2.

To further explore interactions between the most important feature in TE prediction, 3'-UTR sequence length, and other sequence determinants, we analyzed SHAP interaction values in *T. brucei* ( Figure 4). Analysis of the interaction between 3'-UTR length and A-rich tract density ( Figure 4A) and analysis of the reciprocal interaction ( Figure 4B) both revealed a positive correlation with TE, supporting our previous finding, based on a massive parallel reporter assay ^11^. Thus, the new analysis is consistent with the view that A-rich tract dosage and density within 3'-UTRs contribute to TE, but with relatively little impact in UTRs of less than 300 b in length.

### Species-specific contributions of UTRs and codon bias to mRNA abundance

**Figure 5.**
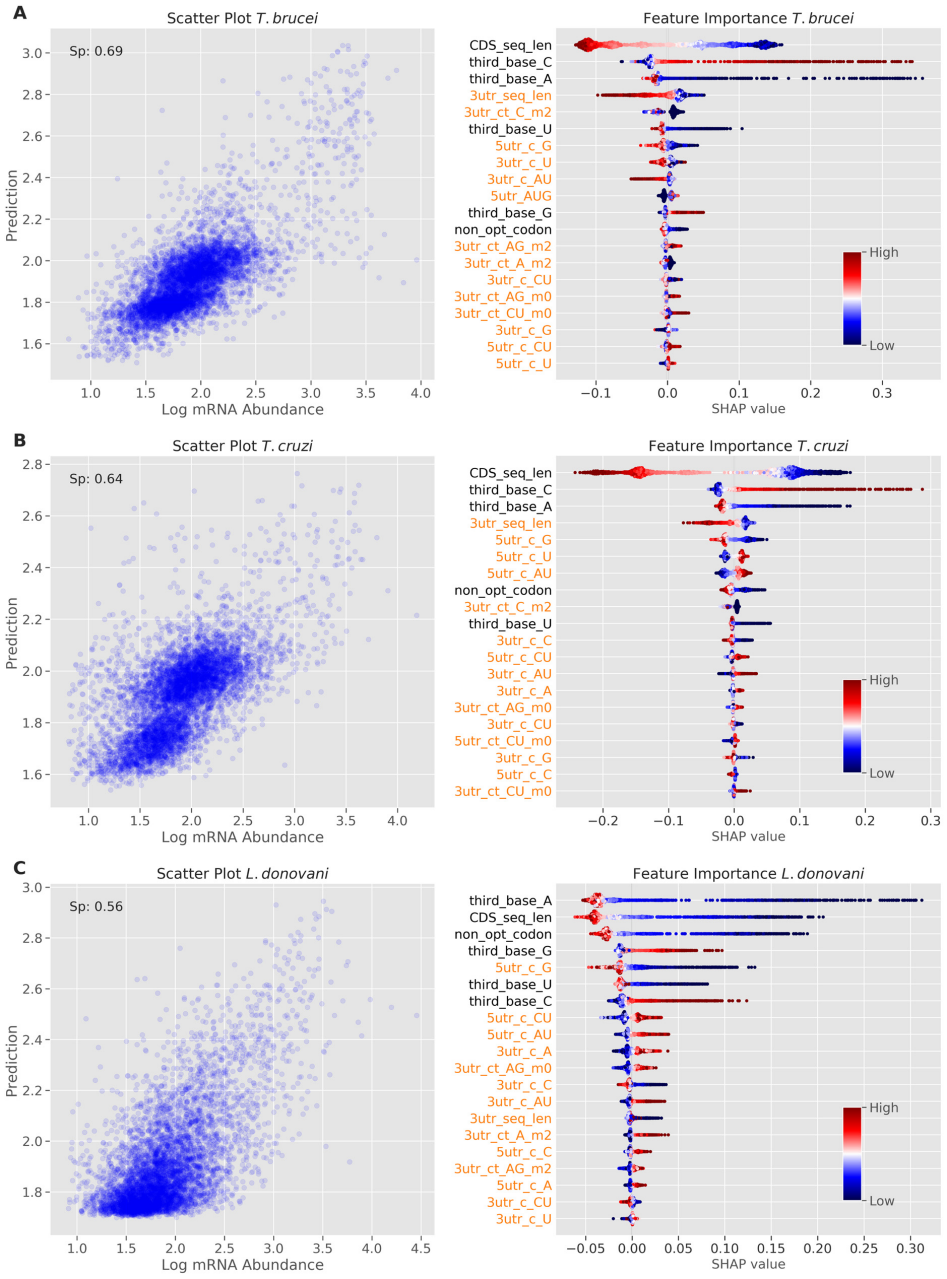
Machine learning models reveal determinants of mRNA abundance. Model performance and feature importance analysis for **A** *T. brucei*. **B** *T. cruzi*. **C** *L. donovani*. The scatter plots on the left show prediction versus measured TPM mRNA abundance values (log10 transformed). Other details as in Figure 2.

Our analyses above suggested that UTRs and codons have pervasive impacts on TE, affecting thousands of mRNA transcripts in each of the trypanosomatids. We next used the machine learning approach to understand the determinants of mRNA abundance. The mRNA abundance models showed a similar or higher predictive power relative to the TE models, with Spearman's rank correlation coefficient values again above 0.55 ( Figure 5). The SHAP values for the top twenty features are shown and this analysis revealed that CDS length, previously reported to be inversely correlated with both mRNA and protein abundance ^9, 29^, and codon usage bias, consistently emerged as strong predictors across all species. As for TE, and as also reported previously ^9, 26^, C or G at the third position were positively correlated with mRNA abundance, while A or U at the third position were negatively correlated with mRNA abundance ( Figure 5). While *T. brucei* ( Figure 5A) and *T. cruzi* ( Figure 5B) registered CDS length as the most important, and inversely correlated feature, this feature ranked second in *L. donovani* ( Figure 5C). UTR sequence features also registered as playing a role, but with relatively lower importance. Notably, the distribution of data points and the ‘long tails’ on some SHAP value plots suggested that codon usage bias has a particularly pronounced impact on the abundance of a subset of mRNAs in each species. Thus, similar to the TE analysis, the machine learning models revealed that both codon usage and UTR features contribute to mRNA abundance, though their relative importance varies both within a species and between species.

As above, we ran the machine learning analysis of mRNA abundance on expression data from insect stage *T. brucei* ^7^, and compared the results ( Figure 3B) to the bloodstream stage analysis ( Figure 5A). Feature importance and their relative contributions to the mRNA abundance predictions, as visualized using SHAP values, were very similar for these two distinct life cycle stages, again consistent with the view that the patterns we observed represented species differences rather than developmental differences within a species.

### The relative contributions of UTRs and codons to expression control

**Figure 6.**
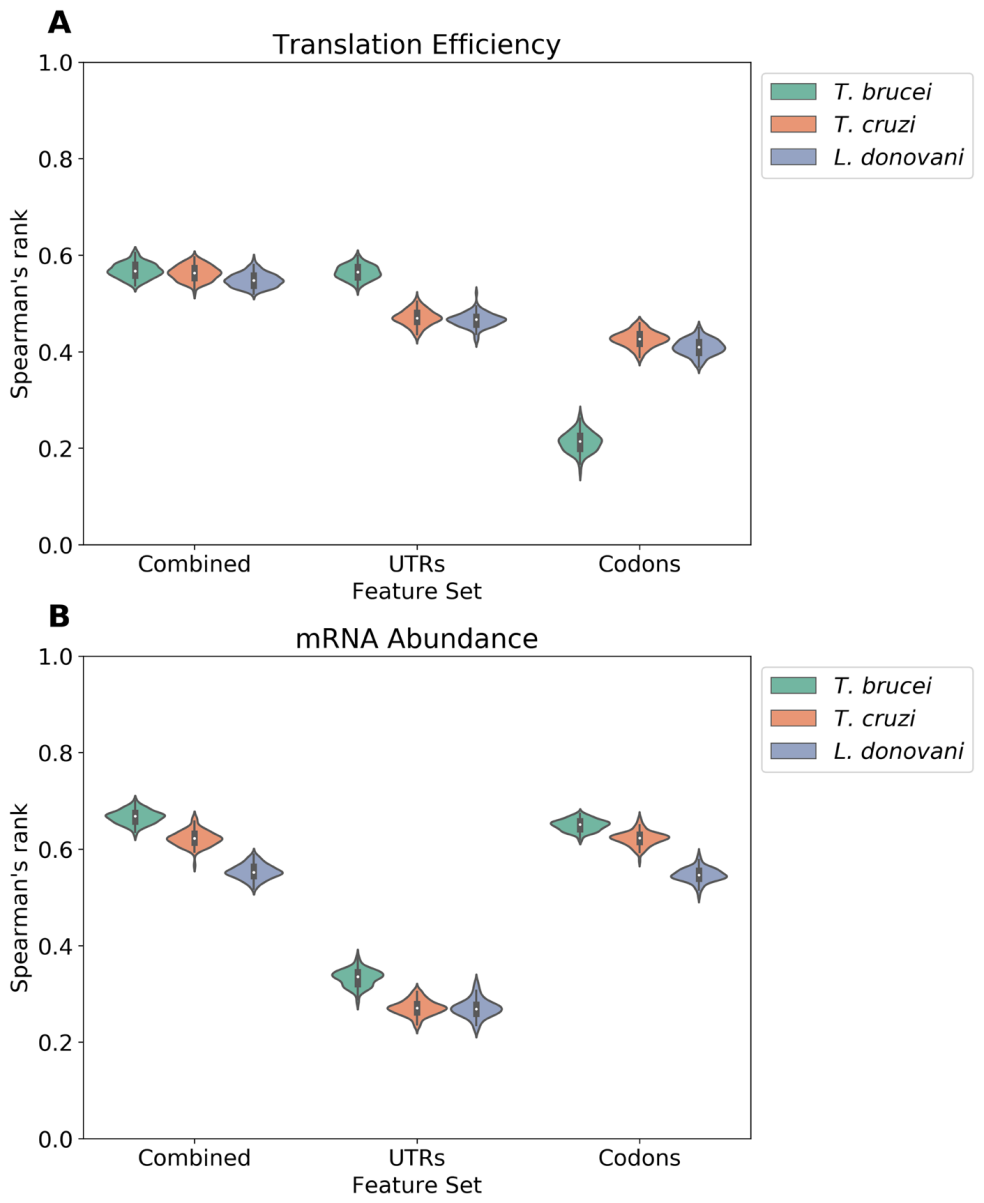
The relative contributions of UTRs and codons to expression control. **A** The violin plot shows the distribution of Spearman's rank correlation coefficients between predicted and observed translation efficiency (TE) in *T. brucei*, *T. cruzi*, and *L. donovani*. Each distribution represents 100 iterations of machine learning models, where each iteration used a random 70/30 train-test split. Models were trained using three distinct feature sets: 3' UTR-derived features only (UTRs), codon-derived features only (Codons), or a combination of both UTR and codon features (Combined). **B** A similar analysis for mRNA abundance predictions, showing the distribution of Spearman's rank correlation coefficients between predicted and observed mRNA levels across 100 iterations with the same feature sets and train-test split methodology as in **A**.

The machine learning models used above indicated substantial differences in terms of the contributions that UTRs and codon usage make to TE and mRNA abundance. To further visualize these differences, we performed model comparisons using 100 independent train-test splits with varying random seeds and different feature sets. The resulting Spearman's rank correlation coefficients were computed and visualized, demonstrating that the models perform with similar results independently of the subset of genes under consideration ( Figure 6). In terms of predicting TE, a comparison of predictions using either UTRs, codons, or both sets of features combined, indicated that the UTR features indeed made the greatest contribution to the predictions in all three trypanosomatids ( Figure 6A); codons make a lesser contribution in *T. brucei*. In terms of predicting mRNA abundance, a similar comparison indicated that codon features make the greatest contribution to the predictions in all three trypanosomatids ( Figure 6B). Thus, our machine learning models indicated that UTRs are strong predictors of ribosome density in trypanosomatids. The results further suggest that codon usage bias makes a greater contribution to mRNA abundance control.

**Figure 7.**
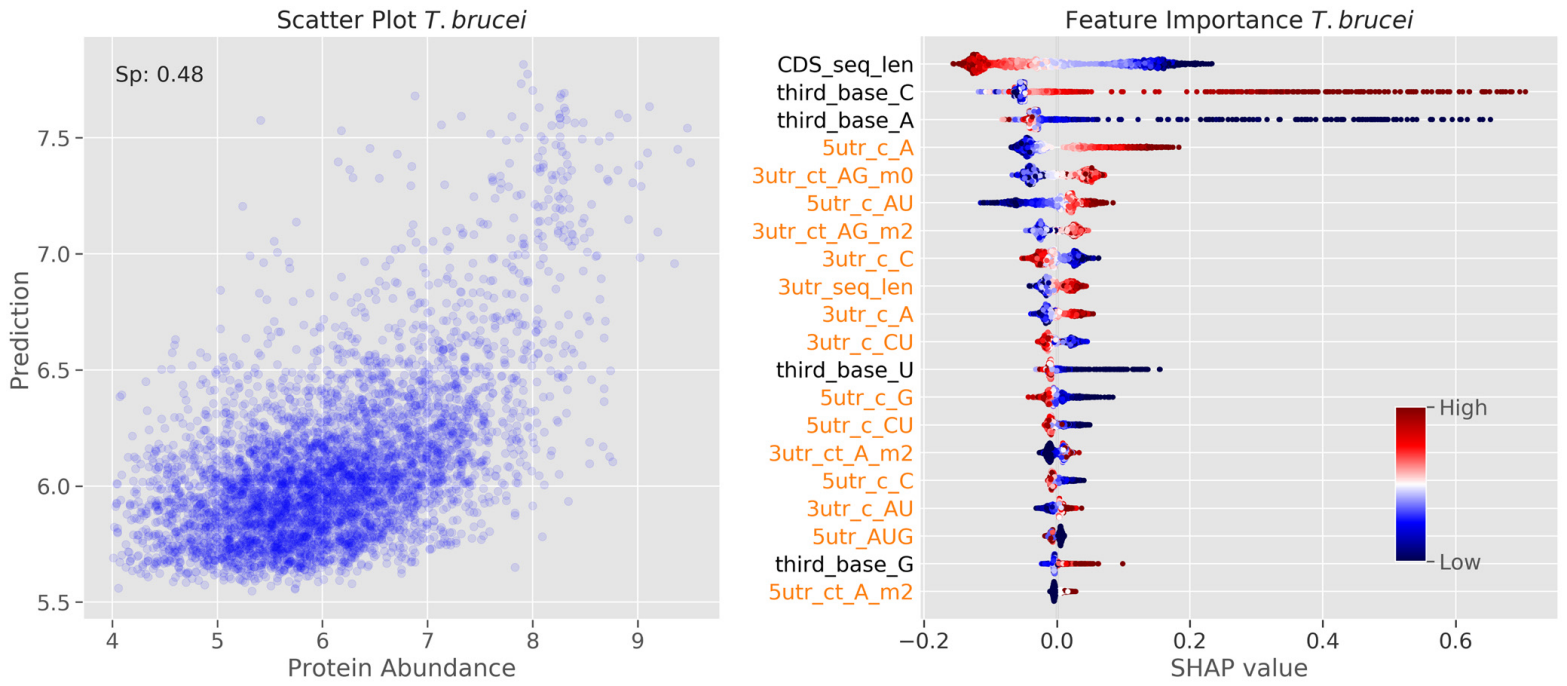
Machine learning models reveal determinants of protein abundance. Model performance (left) and feature importance analysis (right) for bloodstream stage *T. brucei.* The scatter plot on the left shows prediction versus intensity based absolute quantification (iBaq) values (log10 transformed). Other details as in Figure 2.

We finally used the machine learning approach to understand the determinants of protein abundance in *T. brucei*. We anticipated that substantial differences in translation initiation and/or elongation rates, if driven by the sequence features we assessed, would yield a different SHAP profile for protein abundance predictions, relative to the profile we obtained when predicting TE ( Figure 2A). Our reasoning here was that measures of ribosome density reflect both rates of translation initiation and elongation, such that rapid translation could reduce ribosome density, for example. We used a large-scale proteomics dataset ^25^ for this analysis and obtained a Spearman's rank correlation coefficient of 0.48; the resulting SHAP values for the top twenty features are shown ( Figure 7). Rather than 3'-UTR sequence length, which emerged as the most important predictor of TE ( Figure 2A), CDS length registered as the most important, and inversely correlated, predictor of protein abundance; this feature also ranked 1 ^st^ for our mRNA abundance prediction ( Figure 5A), but ranked 18 ^th^ for our TE prediction ( Figure 2A). Finally, and again more consistent with what we observed for our mRNA abundance prediction ( Figure 5A) than with our TE prediction ( Figure 2A), ‘third base’ codon features were assigned higher importance as predictors of protein abundance, again with C or G at the third position positively correlated with abundance ( Figure 7). This observation is consistent with the view that GC3 codons increase translation elongation speed ^12^ and consequently reduce mRNA turnover ^12, 13^. As for the mRNA abundance predictions ( Figure 5A), the ‘long tails’ on the SHAP value plots for ‘third base C’ and ‘third base A’ suggest that these features have a pronounced impact on the abundance of a subset of rapidly translated transcripts and proteins. This may reflect cases where rapid ribosome translocation reduces ribosome density, such that these features can in some cases be poor predictors of the number of protein molecules produced per transcript per unit time.

## Discussion

Building on the massive parallel reporter assay and machine learning approaches described by Trenaman *et al.* (2024) for *T. brucei* ^11^, we explored the impacts of untranslated regions (both 5'-UTRs and 3'-UTRs), and codon usage bias, on translation and mRNA abundance control in three parasitic trypanosomatid species: *T. brucei*, *T. cruzi*, and *L. donovani*. Our comparative predictions revealed conserved *cis*-acting sequences linked to these post-transcriptional controls and also some notable differences.

In this study, we posed three questions. First, what relative contributions do UTRs and codon usage bias make to TE in *T. brucei*? Our models indicated that UTR features make the greater contribution to TE. Our second question was, what relative contributions do these sequences make to mRNA steady-state levels in *T. brucei*? Here, our models indicated that codon usage bias makes the greater contribution. Our third question was, do these sequences make substantial contributions to mRNA steady-state levels and/or TE in the related parasitic trypanosomatids, *T. cruzi* and *Leishmania*? Our models indicated several similarities, but codon usage bias appears to make a greater contribution to TE in both *T. cruzi* and *L. donovani*, while distinct UTR sequences appear to contribute to TE in *Leishmania*.

We find that long 3'-UTRs enriched in low-complexity sequence tracts are predictive of increased translation efficiency, as recently reported in *T. brucei* ^11^, in both *T. cruzi* and *Leishmania*. Specifically, we suggest that the dosage and density of A-rich poly-purine tracts (pPuTs) promote translation in both *T. brucei* and *T. cruzi*, while dosage and density of (A)U-rich tracts may similarly promote translation in *Leishmania*. Notably, we find similar sequence tracts linked to translation efficiency in both 5'-UTRs and 3'-UTRs, A-rich and AG-rich tracts in trypanosomes, for example. This suggests that both 5'-UTRs and 3'-UTRs function similarly, rather than each containing complementary sequences involved in 5' / 3'-UTR base-pairing, for example. AU-rich elements in 3'-UTRs have been described in some detail in other eukaryotes and contribute to both negative and positive control ^14^. Our models linked AU-count to positive control of translation in all three trypanosomatids, although the contributions were relatively weak in *T. cruzi*. Further evidence for conserved mechanisms in eukaryotes comes from a recent study that linked C-rich motifs in human 5'-UTRs to negative control, and AU-rich motifs to positive control ^30^. It has also recently become clear that sequences throughout an mRNA can interact with translation factors to drive cap-recognition and mRNA activation ^31^.

Codon usage is a major determinant of mRNA abundance through translation dependent impacts on mRNA turnover ^12, 13^, and this also appears to be the case in trypanosomatids. The Dhh1 helicase drives this process in yeast by binding slow-moving ribosomes and targeting transcripts with non-optimal codons for degradation ^32^, as does the DDX6 orthologue in humans ^33^. The DHH1 orthologue targets developmentally regulated transcripts in *T. brucei* ^34^, possibly via a similar mechanism. Notably, the impact of favored codons was more readily apparent in *T. brucei* when predicting measures of protein abundance rather than ribosome footprints, and we suggest that this reflects faster ribosome translocation rates. Thus, ‘translation efficiency’ is typically calculated by dividing ribosome footprint read-counts by mRNA read-counts ^7, 28^, but this may more accurately be described as ‘ribosome density’, since both translation initiation rate and ribosome translocation speed contribute to the number of proteins produced per mRNA per unit time.

Our findings indicate that both favored codons, and low-complexity motifs in UTRs in trypanosomatids, co-operate to promote gene expression by recruiting more, faster-translocating ribosomes that reduce mRNA turnover. In terms of the relative contributions of favored codons and UTRs, we suspect that UTRs can evolve more rapidly than codons in a constrained protein coding sequence, such that favored codons primarily contribute to controlling the expression of highly conserved proteins. Additional features impact gene expression control in trypanosomatids, including upstream open reading frames ^28^, alternative polyadenylation ^35^, and long non-coding RNAs ^36, 37^, while gene expression changes also occur in response to developmental and environmental transitions ^38^. The machine learning approaches described here may also help us to probe and understand these even more complex layers of gene regulation.

A major challenge in evaluating UTR function is in identifying regulatory *cis*-elements, which are often distributed along the UTR and act co-operatively or synergistically ^14^. Our machine learning models begin to address this challenge by predicting observed measures of translation efficiency and mRNA abundance in trypanosomatids, also deconvolving the relative contributions of 5'-UTR, 3'-UTR, and codon sequences. For instance, longer 3'-UTRs with a high density of A-rich tracts had a positive impact on TE in *T. brucei*, consistent with the results obtained using a massive parallel reporter assay ^11^. Additionally, our results in relation to *L. donovani* indicated distinct 3'-UTR sequences contributing to TE regulation, compared to the trypanosomes. Since, *trans*-acting RNA-binding proteins determine the function of these *cis*-elements, the next challenge, essential to understanding mechanisms of gene expression control, will be to identify those factors. We conclude that by integrating transcriptome and proteome-wide data with sequence feature analysis, we can identify the *cis*-acting sequences used by trypanosomatids to compensate for their severely restricted capacity for transcription control.

## Ethics and consent

Ethical approval and consent were not required.

## Data Availability

Previously deposited datasets re-processed in this study. BioProject: *T. brucei* gene expression data from BioProject. Accession number PRJNA246300; http://identifiers.org/bioproject:PRJNA246300. BioProject: *T. cruzi* gene expression data from BioProject. Accession number PRJNA260933; http://identifiers.org/bioproject:PRJNA260933. BioProject: *L. donovani* gene expression data from BioProject. Accession number PRJNA495919; http://identifiers.org/bioproject:PRJNA495919. Zenodo: *T. brucei 927* iBAQ protein abundance. https://doi.org/10.5281/zenodo.14923652 ^39^. This project contains the following underlying data: indata2.csv (iBAQ values for protein intensity values in *T. brucei*) Zenodo: decoding-gene-expression. https://doi.org/10.5281/zenodo.14872061 ^40^. This project contains the following underlying data: compare.ipynb (jupyter notebook to reproduce the figure of the paper) TB/ML_DATASET.csv.gz (target variables and features for *T. brucei* analysis) TB/iBAQ_927.csv.gz (target variables for protein abundance for *T. brucei*) TB/stats_* (pre-computed outputs of Machine Learning models for *T. brucei*) TB/viz_dataset.csv.gz (pre computed data of Figure 1 for *T. brucei*) TB_pcf/ML_DATASET.csv.gz (target variables and features for *T. brucei*, procyclic form analysis) TB_pcf/stats_* (pre-computed outputs of Machine Learning models for *T. brucei*, procyclic form) TC /ML_DATASET.csv.gz (target variables and features for *T. cruzi* analysis) TC/stats_* (pre-computed outputs of Machine Learning models for *T. cruzi*) TC/viz_dataset.csv.gz (pre-computed data of Figure 1 for *T. cruzi*) LD /ML_DATASET.csv.gz (target variables and features for *L. donovani* analysis analysis) LD/stats_* (pre-computed outputs of of Machine Learning models for *L. donovani*)) LD/viz_dataset.csv.gz (pre-computed data of Figure 1 for *L. donovani*) Data are available under the terms of the Creative Commons Attribution 4.0 International license (CC-BY 4.0).

## Software availability

Source code available from: https://github.com/mtinti/decoding-gene-expression.

Archived software available from: https://doi.org/10.5281/zenodo.14872061 ^40^.

License: MIT. The code can be executed and reproduced using the MyBinder application badge on the GitHub repository.
