# Supplementary material for: Decoding post-transcriptional gene expression controls in trypanosomatids using machine learning
Source: Wellcome Open Res. 2025 Jul 9;10:173. Originally published 2025 Apr 4. [Version 2] doi: 10.12688/wellcomeopenres.23817.2 (PMC12304876; doi:10.12688/wellcomeopenres.23817.2)
Supplement: Peer review history

# Reviewer report: Reviewer response for version 2

**DOI:** 10.21956/wellcomeopenres.27142.r127541

**Published:** 2025-08-30

**Authors:** Maria Camila Hoyos Sanchez

**Affiliations:** 1 Texas Tech University, Amarillo, Texas, USA

The manuscript by Tinti and Horn makes a valuable contribution to understanding post-transcriptional regulation in trypanosomatids. By applying machine learning to transcriptomic, ribosome profiling, and proteomic datasets, the authors provide important insights into how untranslated regions (UTRs) and codon usage bias shape gene expression across *T. brucei, T. cruzi, and L. donovani*. The study is concise, clearly written and will contribute valuable knowledge on the mechanisms of post-transcriptional regulation to parasitologists.

 Comments:

 1. The authors reduced redundancy by clustering 3′ UTR sequences and removing those with >40% identity, which addresses similarity at the UTR level. In *T. cruzi*, some large gene families such as GP63 have been shown in a recent 2025 study to display variability in their 3′ UTRs depending on whether the genes are located in the core or disruptive compartments of the genome. Did the authors check whether the UTR-based filtering also effectively reduced redundancy at the CDS level in these cases? Clarifying this point would help to assess whether CDS-level conservation could partly underlie the stronger contribution of codon usage bias observed in *T. cruzi*.

 2. The machine learning framework is described clearly. The dataset was partitioned into a 70% training set and a 30% test set using 3-fold cross-validation. To help readers fully assess model robustness, it would be useful to report the number of genes retained for each organism after filtering. In addition, were both training and test splits stratified by expression levels? Even after low-abundance filtering, expression levels can vary considerably, and high-abundance genes could dominate signal. Explaining whether the splits preserved a balanced distribution across expression levels would enhance confidence that the reported correlations reflect model performance across the full dynamic range of expression levels.

 3. The model for protein abundance in *T. brucei* yielded a lower correlation (Sp: 0.48) compared to the stronger performance of the TE and mRNA models. A short discussion of this difference would be valuable. Could this reduced performance reflect biological complexity, such as post-translational regulation of proteins, or is it more likely influenced by technical limitations of proteomic quantification (e.g., peptide detectability, dynamic range)? Clarifying this point would help the audience interpret the extend and limitations of the modeling framework.

Is the work clearly and accurately presented and does it cite the current literature?

Yes

If applicable, is the statistical analysis and its interpretation appropriate?

Yes

Are all the source data underlying the results available to ensure full reproducibility?

Yes

Is the study design appropriate and is the work technically sound?

Yes

Are the conclusions drawn adequately supported by the results?

Yes

Are sufficient details of methods and analysis provided to allow replication by others?

Yes

Reviewer Expertise:

Parasite genomics, bioinformatics, evolution of gene families.

I confirm that I have read this submission and believe that I have an appropriate level of expertise to confirm that it is of an acceptable scientific standard.

# Reviewer report: Reviewer response for version 2

**DOI:** 10.21956/wellcomeopenres.27142.r127167

**Published:** 2025-07-28

**Authors:** Pablo Smircich

**Affiliations:** 1 Sección Genómica Funcional, Facultad de Ciencias, Universidad de la República, Montevideo, Uruguay; 2 Bioinformatics lab, Genomics Department, Instituto Clemente Estable, MEC, Montevideo, Uruguay

I thank the authors for addressing one of my main concerns and believe the manuscript is suitable for indexing as it is. However, I still think the final version would benefit from including the authors’ thoughts on the rest of my comments on version 1.

Is the work clearly and accurately presented and does it cite the current literature?

Yes

If applicable, is the statistical analysis and its interpretation appropriate?

Yes

Are all the source data underlying the results available to ensure full reproducibility?

Yes

Is the study design appropriate and is the work technically sound?

Partly

Are the conclusions drawn adequately supported by the results?

Yes

Are sufficient details of methods and analysis provided to allow replication by others?

Yes

Reviewer Expertise:

Molecular parasitology, genomics and bioinformatics

I confirm that I have read this submission and believe that I have an appropriate level of expertise to confirm that it is of an acceptable scientific standard.

# Reviewer report: Reviewer response for version 1

**DOI:** 10.21956/wellcomeopenres.26269.r121928

**Published:** 2025-05-10

**Authors:** Pablo Smircich

**Affiliations:** 1 Sección Genómica Funcional, Facultad de Ciencias, Universidad de la República, Montevideo, Uruguay; 2 Bioinformatics lab, Genomics Department, Instituto Clemente Estable, MEC, Montevideo, Uruguay

In the manuscript by Tinti and Horn, the authors explore how specific gene features, such as codon usage bias and untranslated region (UTR) length and sequence composition, influence gene expression in model trypanosomatids. They examine the extent to which these characteristics affect translation efficiency (TE), steady-state mRNA levels, and protein abundance, revealing species-specific differences in their relative contributions. To do this, they apply machine-learning models to transcriptomic, Ribo-seq, and proteomic datasets. Based on their findings, the authors propose a model for translation dynamics that accounts for the roles of initiation rates and ribosome translocation speeds. These findings are certainly valuable for the trypanosomatid research community.

 Comments:

 1.    The authors define UTRs as: “for each gene, its 3’ UTR was defined as the intergenic region between its stop codon and 200 nucleotides upstream of the start codon of the downstream gene”. Given that conclusions about UTR length and the extraction of key sequence features for the analysis rely on this definition, it would be helpful to further explain the rationale behind this criterion. 

 2.    Did the authors account for the potential effect of large gene families and the biases they may introduce in the analysis?

 3.    In Figure 2, T. cruzi displays a bimodal distribution of SHAP values for 3′ UTR lengths, whereas T. brucei and Leishmania show continuous distributions with long tails. Could the authors comment on this feature? For example, is there a 3′ UTR length threshold in T. cruzi that differentiates high and low TE genes, potentially explaining the bimodality?

 4.    The x-axis scales in the beeswarm plots differ between species and figures. In Figure 2, it would be important either to preserve a consistent scale across species for direct comparison or to comment or to highlight that features for Leishmania appear less predictive, as suggested by the lower SHAP values. Additionally, it is notable that for mRNA abundance, SHAP values only reach 0.4, while for TE they approach 0.8 in some cases. A brief discussion of this difference in predictive power would be valuable.

Is the work clearly and accurately presented and does it cite the current literature?

Yes

If applicable, is the statistical analysis and its interpretation appropriate?

Yes

Are all the source data underlying the results available to ensure full reproducibility?

Yes

Is the study design appropriate and is the work technically sound?

Partly

Are the conclusions drawn adequately supported by the results?

Yes

Are sufficient details of methods and analysis provided to allow replication by others?

Yes

Reviewer Expertise:

Molecular parasitology, genomics and bioinformatics

I confirm that I have read this submission and believe that I have an appropriate level of expertise to confirm that it is of an acceptable scientific standard, however I have significant reservations, as outlined above.

# Reviewer report: Reviewer response for version 1

**DOI:** 10.21956/wellcomeopenres.26269.r121924

**Published:** 2025-05-07

**Authors:** Antonio Estevez

**Affiliations:** 1 Instituto de Parasitología y Biomedicina López - Neyra, Granada, Spain

Following their recent and elegant work on genome-scale parallel reporter assays and 3′-UTR-seq profiling in *T. brucei*, Tinti and Horn here explore the effects of UTRs and codon usage bias on translation and mRNA abundance. By integrating RNA-seq, ribosome profiling, and proteomic datasets with sequence feature analysis and clustering, the authors neatly dissect the relative contributions of UTR features and codon usage to translation efficiency and mRNA levels in *T. brucei*, *T. cruzi*, and *L. donovani*. They uncover conserved features as well as interesting differences among the three organisms.

 The work is concise, well presented, and thoughtfully discussed. The results are convincing and will greatly contribute to our understanding of how trypanosomatids regulate gene expression in the absence of transcriptional control. They also offer a solid basis for developing new approaches to identify trans-acting regulatory factors.

 I have only minor comments and suggestions for improvement:

 1) *L. donovani* generally has much longer 3′-UTRs compared to trypanosomes (about twice as long). Is this also true for other *Leishmania* species? Why might trypanosomes have shorter UTRs? Could the authors speculate on this?

 2) The authors note that codons contribute less to translation efficiency in *T. brucei* than in the other two species (Fig. 6). Could there be a biological explanation for this (for example, the low G/C content at the third codon position)?

 3) The SHAP value plots (Figures 5 and 7) show intriguing ‘long tails’. The authors suggest this may indicate that codon usage bias has an especially strong impact on the abundance of a subset of mRNAs (Fig. 5) or proteins (Fig. 7) in each species. Could this be explored further? For instance, is there overlap between the mRNA subset in Fig. 5 and the protein subset in Fig. 7? Are the corresponding transcripts/proteins conserved across the three species? Are these proteins ‘special’ in some way (e.g., are they mostly ribosomal, or highly abundant, or enriched for particular GO terms)?

Is the work clearly and accurately presented and does it cite the current literature?

Yes

If applicable, is the statistical analysis and its interpretation appropriate?

Yes

Are all the source data underlying the results available to ensure full reproducibility?

Yes

Is the study design appropriate and is the work technically sound?

Yes

Are the conclusions drawn adequately supported by the results?

Yes

Are sufficient details of methods and analysis provided to allow replication by others?

Yes

Reviewer Expertise:

RNA-binding proteins, regulation of gene expression

I confirm that I have read this submission and believe that I have an appropriate level of expertise to confirm that it is of an acceptable scientific standard.
